# Failing Others’ Expectations: Negative Emotions and Behavior Change in Daily Life

**DOI:** 10.1007/s42761-026-00365-w

**Published:** 2026-04-23

**Authors:** Madhuri Kashyap, Jiayan Chang, Chunlei Lu, Weijian Li, Yang Hu, Xiaolin Zhou, Hongbo Yu

**Affiliations:** 1 Department of Psychological and Brain Sciences, University of California Santa Barbara, Santa Barbara, CA 93106-9660 USA; 2 Institute of Psychological and Brain Sciences, Zhejiang Normal University, Jinhua, China; 3 School of Psychology and Cognitive Science, Shanghai Key Laboratory of Mental Health and Psychological Crisis intervention, East China Normal University, Shanghai, China

## Abstract

Social expectations are a ubiquitous part of human experience, yet little is known about the emotions associated with failing to fulfill others’ social expectations. In two studies, we examined the effects of failing to fulfill others’ social expectations on individuals’ emotions, and assessed how situational factors moderate these effects. Study 1 aimed to establish the relationship between social expectation violation and negative affects in ecologically valid everyday contexts using daily diary methods. We found that in both Chinese and American college student samples, negative affects scaled with the degree to which one failed to fulfill a social expectation (i.e., violation). Moreover, the perceived legitimacy and strength of an expectation modulated the relationship between expectation violation and negative affect. Study 2 showed that the participants in Study 1 systematically overestimated the legitimacy and strength of the social expectations they reported relative to independent raters who evaluated the social expectations from a third-person perspective. Importantly, overestimation of the legitimacy of social expectations led to significantly higher self-reported negative affects. Taken together, we demonstrated the critical role of failing to meet social expectations in individuals’ negative social emotions in two cultural contexts and pinpointed important modulating factors of such effects. These results have implications for interventions aiming to alleviate the impacts of social expectations on emotional well-being.

**Supplementary Information:**

The online version contains supplementary material available at 10.1007/s42761-026-00365-w.

## Introduction

Our social environments are uncertain, and social learning often operates through social expectations—beliefs about how others will behave—that serve as reference points guiding affective and behavioral responses (Baker et al., 2017; McNulty & Karney, 2002). Conforming to perceived expectations can have harmful or beneficial consequences (Afifi & Metts, 1998; Caspar et al., 2016; Nook et al., 2016). Although expectations have been extensively studied from the expectation holder’s perspective (McNulty & Karney, 2004; Villano et al., 2020), less is known about what happens emotionally when people believe they have failed to meet others’ expectations, from the perspective of the expectation recipient.

We focus on idiosyncratic, relationship-specific expectations rather than broad social norms. Norms (e.g., fidelity, honesty) reflect shared cultural rules and shape behavior (Van Kleef et al., 2015), but they do not capture the expectations that arise from a dyad’s unique history and needs. Such person-specific expectations can predict emotional experience and relationship functioning beyond generalized norms (Miller & Carlson, 2016). We also distinguish expectations from desires: expectations reflect realistic anticipations grounded in relational history, whereas desires reflect ideal wishes (Holmes, 2002). Because expectations are perceived as more warranted, their violation typically carries stronger emotional consequences than unmet desires (Fletcher & Simpson, 2000). Throughout, we treat expectations primarily as descriptive beliefs about what a partner is likely to do, which are especially relevant for everyday interaction (Reno et al., 1993).

Emotions help track the discrepancy between expected and actual outcomes and can function as “error signals” that guide learning or expectation updating (Bhatia et al., 2019; Ding & Liu, 2022; Schultz, 2015; Shepperd & McNulty, 2002; Villano et al., 2020, 2023). In relationships, expectations are dynamic and relationship-dependent. Therefore, the same event can elicit different emotions across relational contexts (Bugental, 2000; Earp et al., 2021). Expectation Violation Theory (EVT) has primarily examined the holder’s reactions (Burgoon, 1993; Burgoon & Hale, 1988). While prior work on social expectation has primarily focused on the perspective of the expectation holder, such as the Expectation Violation Theory (EVT) (Burgoon, 1993; Burgoon & Hale, 1988), here we emphasize the perspective of the expectation recipient, who infers what others expect and evaluates their own behavior relative to those inferred standards. When people perceive they have violated a partner’s expectation, they may experience negative self-conscious affect (e.g., guilt) and motivation to repair or change behavior (Battigalli & Dufwenberg, 2007; Theriault et al., 2021).

We propose that the impact of perceived violations depends on legitimacy (how warranted the expectation is) and strength (how strongly it is held) of the expectation, and closeness to the expectation holder. Legitimacy and strength should modulate emotional and motivational responses, analogous to gating in learning: deviations matter more when they reflect meaningful contingencies rather than noise (Kawato & Samejima, 2007; Li et al., 2011; McGuire et al., 2014). Closeness should also amplify consequences because expectations from close partners are more central and personally consequential.

We also explore cultural variability. Emotions and social expectations differ across cultures (Mesquita, 2022; Tsai et al., 2006), and cultures vary in how interpersonal obligations are moralized (Miller et al., 1990; Miller & Bersoff, 1992). Stronger moralization of relational obligation may predict stronger guilt and reparative motivation following perceived violations.

A further issue is whether recipients’ appraisals of the expectations are biased. Because people are embedded in relationships, perceived expectation strength and legitimacy may be shaped by emotional involvement or motivated reasoning (Burgoon & Hale, 1988; Higgins, 1987). Such bias may be especially consequential in close relationships and may vary across cultural contexts that emphasize obligation versus autonomy (Markus & Kitayama, 1991; Oishi & Sullivan, 2005).

In the present research, we test how perceived expectation violations relate to negative affect and motivation from the recipient perspective in two studies. Study 1 uses daily diaries to examine links between perceived violations and guilt/depression and whether legitimacy, strength, and closeness moderate these effects, comparing U.S. and Chinese participants. Study 2 tests whether recipients’ situational evaluations systematically deviate from third-party ratings and whether such discrepancies predict stronger negative affect and motivation for change.

## Study 1

Study 1 uses a daily diary approach to capture expectations participants experience in their lives, the extent to which they believe they fulfilled them, and associated emotional and behavioral responses. Building on a pilot vignette-based study (Supplementary Materials: 1. Pilot Study), we preregistered that (1) negative emotions and depressive mental states will correlate with lower perceived fulfillment, and (2) perceived strength, legitimacy, and social closeness will modulate these relationships. To explore cultural variability and generalizability of our findings, we recruited both Chinese and American participants.

### Method

#### Study Overview

In Study 1, we sought to establish the relationship between failing to fulfill social expectations and negative affect in people’s everyday life, by using an ecologically valid method – daily diary (Greenaway et al., 2018). With this method, we aimed to capture the nuances of the perception of and reactions (emotional and behavioral) to social expectation, and to provide a more ecologically valid basis for generalizability to daily experience. Participants were given a daily diary survey to report expectations they were facing in their own lives, and several affects they experienced related to the expectation. Another aim of Study 1 is to examine whether and how failing to fulfill social expectations contributes to depressive mental states in college students’ everyday life. Some prior research has shown that family expectations regarding academic performance and career trajectory is a source of students’ stress and negative experience and a risk factor of poor mental health condition (Covarrubias et al., 2020; Deb et al., 2015; Nguyen et al., 2020; Shen et al., 2014). Study 1 also examined how situational factors, such as the strength and legitimacy of the expectation and the social closeness with the source of the expectation, could modulate the relationship between social expectation violation and negative affects and depressive mental states. A pilot study using a vignette-based task demonstrated that negative affect (e.g., guilt) due to failing to fulfill others’ expectation positively scaled with how strong and legitimate one perceives the expectation to be (see Supplementary Materials: 1. Pilot Study). We therefore preregistered two main hypotheses: (1) negative emotions and mental states (e.g., guilt, depression, etc.) would be negatively correlated with the extent to which an expectation is fulfilled, (2) situational factors (strength, legitimacy, and social closeness) would modulate the above relationships. To explore cultural variability and generalizability of our findings, we recruited both Chinese and American participants.

#### Participants

U.S. American (*N* = 201) and Chinese (*N* = 200) undergraduate students were recruited through Prolific or university-based recruitment programs. Of these, 66 participants (50 American and 16 Chinese participants) were excluded from the final analysis due to failing the attention check questions, or for not reporting any expectations. Participants were instructed to create a unique ID that would connect their anonymized daily diary responses. We also excluded any survey responses where this field was left blank. The final sample consisted of 335 participants (American: *N* = 151, 123 female participants, *M*_age_ = 20.1, *SD*_age_ = 4.1; Chinese: *N* = 184, 139 female participants, *M*_age_ = 20.9, *SD*_age_ = 2.9). The data collection and analysis plan for the American sample was formally pre-registered (https://aspredicted.org/VCH_BD8). The Chinese data was collected and analyzed in a consistent manner as the American sample. All participants provided written consent electronically before the start of the study. The protocol is approved by the Human Subjects Committee of University of California Santa Barbara (protocol number: 14-25-0266).

#### Procedure

The study consisted of two phases: an online intake phase and a mobile (daily diary) phase. Participants registered either online or through a university-based platform and completed the online intake phase first. The survey asked participants to recall a social expectation they had experienced in the past week and answer questions pertaining to it. They were then guided to the platform (*SurveySignal*) where they could sign-up for the mobile phase (Hofmann & Patel, 2015). Once the sign-up was complete and participants received confirmation, they were prompted to return to the online survey and complete some individual differences questionnaires presented in a randomized order (see below for detail), as well as some demographic information.

Starting the day after the online intake phase, participants would receive a daily survey via a message on their phones for five consecutive days. The messages were sent at a random time between 18:00 to 21:00 in the time zone they had chosen during sign-up. The daily survey was similar to the expectation-related questions they completed during the online intake phase and asked them to recall if they felt like someone had expected something from them since the last survey. If the participant reported that they experienced at least one expectation, they would then be asked to describe one of the most noticeable expectations, and answer a few questions about the properties of the expectation (e.g., strength, legitimacy, etc.), the degree to which the participants fulfilled the expectation, the source (e.g., parents, classmates, etc.) and type (e.g., academic performance, relationship) of the expectation, and the participants’ emotional and behavior change this expectation (see Measures below for details).

#### Measures

#### Predictor

The primary predictor variable in this study was participants’ self-reported degree of fulfillment of an expectation (“To what extent did you fulfill the expectation you just described?”). Fulfillment of the expectation is measured on a 5-point Likert scale (1 – *Not at all*, 5 – *Completely).*

#### Dependent Measures

Our dependent variables of guilt and depression were measured using an analogue slider scale, ranging from 0 (Not at all) − 100 (Extremely). Participants were asked to rate the extent to which they felt the emotions/mental states when they contemplated the expectation they had previously described and the extent to which they had fulfilled it (“Considering this expectation and how much you have fulfilled it, to what extent do you feel the following”). Behavior change was measured using a 5-point Likert scale with the wording “To what extent do you want to (or have already) alter your behavior in order to fulfill the expectation?” (1 – *Not at all;* 5 – *Extremely*). Results of other exploratory measures are reported in Supplementary Materials: 2.2 Other dependent measures from daily diary.

#### Situational Factors

The primary situational factors measured were legitimacy and strength of expectation, as our pilot studies have demonstrated the association between legitimacy and strength with emotional and behavioral consequences of failing to fulfill a social expectation. Strength and legitimacy were measured as in the pilot studies (strength: “How strong is the expectation?”, 5-point Likert scale; legitimacy: composite score of reasonableness, warrantedness, and justifiability, also on a 5-point Likert scale). Recent work has suggested that the moral wrongness of an action is contingent on the specific relationship the act was committed within (Earp et al., 2021). Therefore, we measured closeness to the source of the expectation by asking participants to indicate how socially close they felt between themselves and the source of the expectation they had in mind. Closeness was measured using the Inclusion of Other in Self (IOS) scale which is a single-item, pictorial measure of closeness (1 – *Not at all close*, 5 – *Very close*; Aron et al., 1992; Country-level differences in these situational factors were reported in Supplementary Materials: 2.4 Situational factors as a function of country).

### Analysis

We first examined the content or nature and sources of these expectations. When evaluating the expectation they reported, participants indicated from whom the expectation came by choosing one from the following relationships: parents, other family members, classmates/coworkers, romantic partner, friends, supervisor/teacher, yourself, supernatural/higher power, society, and other. They also indicated in what categories the expectation fell, for which they could choose any combination of the following: academic performance, career choice, career achievement, relationship investment, religious/spiritual duty, social norms, physical appearance/health, financial benefit, and other. Each source and category were coded as 1 if participants checked them for a given expectation, and 0 otherwise. We used general linear mixed effects models to analyze the data (assuming Poisson distribution of the dependent variables), including participants’ country (American vs. Chinese) as the fixed effect predictor, demographic variables as control variables, and participant ID as a random intercept factor.

We preregistered using linear mixed effects models to test our primary hypotheses. To test our first hypothesis, we examined the effect of expectation fulfillment on our key DVs (i.e., guilt, depression, behavior change), and explored whether this effect is consistent across participants’ groups (i.e., country). To this end, we estimated linear mixed effect models where the key DVs were included as the outcome variable, and fulfillment, country, as well as their interaction were included as the primary predictors. Participant ID was included as a random effect, and fulfillment was also treated as a random slope. To investigate the effects of situational factors of expectations (i.e., perceived legitimacy, strength, etc.) on the relationship between fulfillment and the key DVs, we ran a new set of models similar to the above one, with the addition of the main effect of the situational factors and their interaction with fulfillment. We estimated a separate model for each situational factor.

Upon inspecting the data, we found that some of our outcome variables were non-normally distributed. We report the results of our pre-registered analysis here but also conducted Bayesian mixed effects models as a robustness check of our findings (see Supplementary Materials: 2.3 Bayesian Robustness Check). The results of the Bayesian regression replicate the regression models reported here.

The expectation-related questions in the intake and in the daily diary surveys were analyzed separately as the former is a recall task in which participants recount an expectation they experienced in the two weeks prior to filling out the survey. However, the latter is more similar to a momentary assessment as participants report on an expectation they experienced on the same day as the survey. Below, we report the results from the daily diary data which are generally replicated in the intake data (see Supplementary Materials: 2.1 Intake Data Analysis for full intake data analysis). All analyses were conducted using R Statistical Software (v4.1.2; ).

## Results

### Power Analyses

We conducted post-hoc power analysis for multilevel analyses using Monte Carlo simulations (iterations = 1,000), given our sample size and design (*N* = 335, repeated measures). We had sufficient power (observed power > 0.80) to detect one of the two significant slopes of country and degree of fulfillment. The interaction between the degree of fulfillment and country (American or Chinese) was underpowered (observed power = 0.45).

We ran similar power analyses for our hypotheses centered on the situational factors of legitimacy, strength, and closeness. We had sufficient power to detect the main effects of each as well as the interaction between situational factors and fulfillment but were similarly underpowered in detecting the interactions between situational factors and country (observed power = 0.36), and the three-way interaction between situational factors, fulfillment, and country (observed power = 0.44).

For these underpowered effects, we conducted sensitivity analysis to estimate the minimum detectable effect size (MDES) with 0.80 power, given our sample size, and model specifications. We found that our models were adequate at detecting the effects found (MDES > 0.025), but these interaction results should be interpreted with caution.

### Descriptive Patterns

Chinese participants were more likely than American participants to report having encountered an expectation in the period sampled. Among reported expectations, Chinese participants more often described academic achievement and relationship investment, whereas American participants more often described norm-related expectations. Other expectation categories did not differ meaningfully between groups. For sources of expectation, Chinese participants more frequently reported classmates; other sources showed no clear group differences. For detailed descriptive analysis, please see Supplementary Materials: 2.6 Descriptive Statistics and Figure S3.

### Hypothesis-Driven Analyses

Below, we organize the results of hypothesis-driven analyses according to the three primary dependent variables: guilt, depression, and behavior change.

#### Guilt

Supporting our hypothesis , we found that guilt was negatively associated with the degree of fulfillment of the expectation (main effect of fulfillment: *B* ± *S.E.* = -6.668 ± 1.158, 95% CI = [-8.947, -4.415], *b* =-0.302, *t* = -5.706, *p* < .001). We also found a marginally significant effect of country (*B* ± *S.E.* = 15.424 ± 8.166, 95% CI = [-0.439, 31.535], *b* = 0.235, *t* = 1.889, *p* = .06) and a marginally significant interaction between country and fulfillment on guilt (*B* ± *S.E.*= -3.490 ± 1.958, 95% CI = [-7.357, 0.309], *b* = -0.201, *t* = -1.783; *p* = .075), such that American participants tended to report more guilt and their self-reported guilt was more sensitive to degree of fulfillment. We note that these two effects are not statistically significant and should be interpreted with caution.

**Fig. 1:**
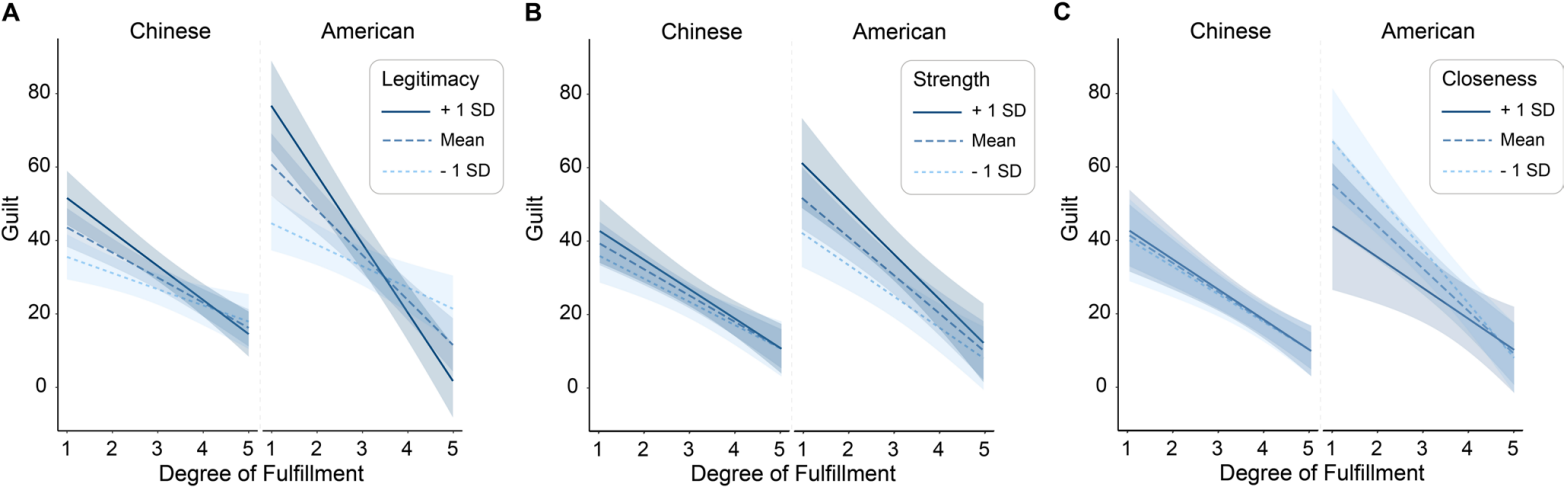
Effects of expectation fulfillment on guilt, modulated by situational factors - legitimacy of expectation (**A**), strength of expectation (**B**), and closeness to the source of expectation (**C**)

Pivoting to the situational factors, we found a significant main effect of perceived legitimacy such that overall if an expectation was perceived as more legitimate, it was associated with more guilt (*B* ± *S.E.*= 10.343 ± 2.906, 95% CI = [4.653, 15.995], *b* = 0.352, *t* = 3.559; *p* < .001) (Fig. 1A). There was also a significant two-way interaction between fulfillment and legitimacy such that failing to fulfill a more legitimate expectation was associated with higher self-reported guilt (*B* ± *S.E*.= -2.405 ± 0.892, 95% CI = [-4.137, -0.657], *b* = -0.566, *t* = -2.647, *p* = .007). We also found a significant country-by-legitimacy two-way interaction (*B* ± *S.E*.= -11.898 ± 4.671, 95% CI = [2.801, 21.028], *b* = 0.71, *t* = -2.695, *p* = .011), such that American participants were more sensitive to legitimacy in their guilt responses. Moreover, this two-way interaction was most pronounced at lower levels of fulfillment of expectations, as evidenced by a three-way interaction (*B* ± *S.E*.= -3.990 ± 1.431, 95% CI = [-6.774, -1.202], *b* = -0.950, *t* = -2.788, *p* = .005). In other words, legitimacy of expectation is a stronger modulator in the American sample than in the Chinese sample.

We found no significant main effect of strength or strength-by-fulfillment interaction on guilt in the daily diary data (Fig. 1B) (see Supplementary Materials: 2.1 Intake Data Analysis for the results based on the intake data).

Perceived closeness to the source of the expectation interacted with country such that American participants reported more guilt when the source of the expectation was socially distant, while Chinese participants expressed greater guilt when failing to fulfill expectation from closer others (*B* ± *S.E*.= -11.861 ± 6.020, 95% CI = [-23.788, 0.069], *b* = -0.569, *t* = -1.970, *p* = .049) (Fig. 1C). The closeness-by-fulfillment interaction was not significant.

#### Depression

The degree of fulfillment had a significant negative effect on self-reported depression, such that a less fulfilled expectation was associated with more depression across countries (*B* ± *S.E.*= -4.40 ± 1.02, 95% CI = [-6.46, -2.42], *b* = -0.217, *t* = -4.304; *p* < .001). We also observed that American participants were more likely to report feelings of depression in comparison to their Chinese counterparts (*B* ± *S.E.*= 21.22 ± 7.94, 95% CI = [5.74, 36.76], *b* = 0.352, *t* = 2.67, *p* < .001). There was also a significant interaction between fulfillment and country (*B* ± *S.E.*= -3.96 ± 1.75, 95% CI = [-7.38, 0.57], *b* = -0.249, *t* = -2.268, *p* = .025) such that American participants’ feelings of depression were more sensitive to degree of fulfillment than Chinese participants.

**Fig. 2:**
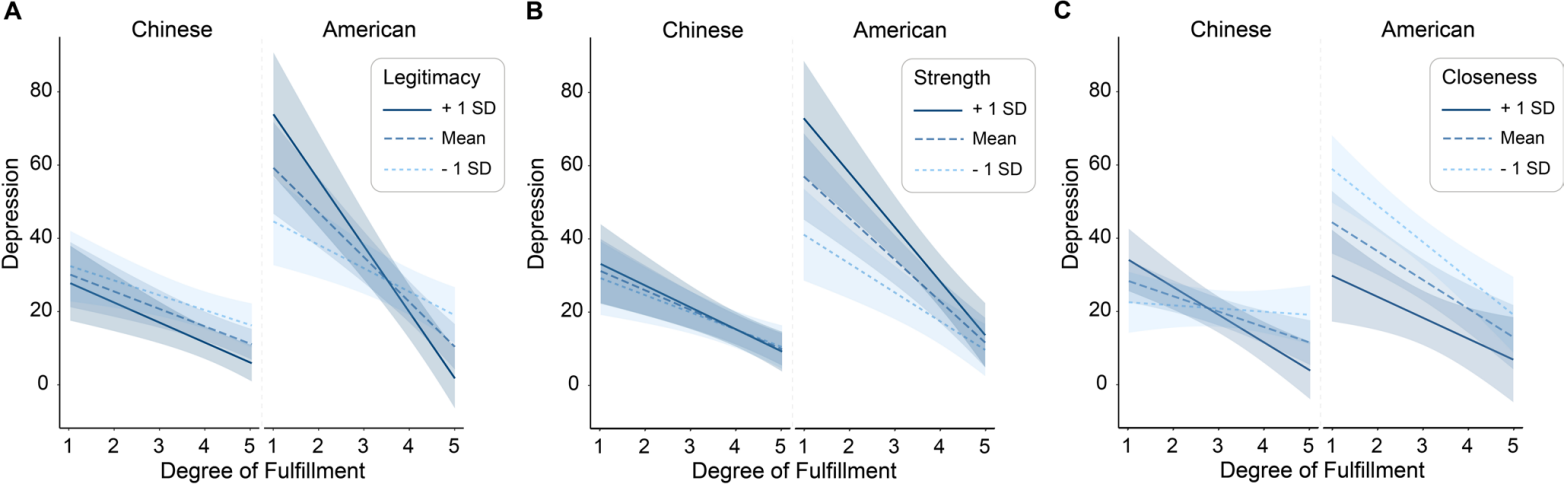
Effects of expectation fulfillment on depression, modulated by situational factors - legitimacy of expectation (**A**), strength of expectation (**B**), and closeness to the source of expectation (**C**)

Perceived legitimacy did not significantly influence depression across country or fulfillment but did interact with country such that American participants were more sensitive to legitimate expectations (*B* ± *S.E*.= 18.429 ± 4.791, 95% CI = [9.065, 27.839], *b* = 1.2, *t* = 3.847, *p* < .001). There was also a significant three-way interaction between legitimacy, fulfillment, and country on feelings of depression (*B* ± *S.E*.= -4.263 ± 1.224, 95% CI = [-6.664, -1.879], *b* = -1.108, *t* = -3.482, *p* < .001) such that American participants were more sensitive to more legitimate expectations when these expectations were farther away from being fulfilled (Fig. 2A).

Perceived strength did not have a significant main effect on depression in the daily diary data (see *Supplementary Materials: 2.1. Intake Data Analysis* for the results based on the intake data), but we found an interaction between country and strength (*B* ± *S.E*.= 15.241 ± 5.796, 95% CI = [3.983, 26.669], *b* = 1.034, *t* = 2.63, *p* = .01) such that stronger expectations were more likely to incite greater depression for the American participants (Fig. 2B).

We found a main effect of perceived closeness (*B* ± *S.E*.= 6.048 ± 2.771, 95% CI = [0.586, 11.423], *b* = 0.276, *t* = 2.183, *p* = 0.029) such that failing to fulfill expectations from closer social partners elicited greater depression in general. There was a significant two-way interaction between perceived closeness and fulfillment (*B* ± *S.E*.= -2.54 ± 0.788, 95% CI = [-4.082, -1.011], *b* = -0.59, *t* = -3.23, *p* < 0.01) such that feelings of depression were more sensitive to fulfillment when the source of the expectations was closer to the participant. However, this two-way interaction between closeness and fulfillment was not replicated in our Bayesian robustness analyses (see Supplementary Materials: 2.3 Bayesian Robustness Check). There was also a significant interaction between closeness and country (*B* ± *S.E*.= -17.65 ± 4.46, 95% CI = [-26.32, -8.92], *b* = -0.95, *t* = -3.96, *p* < 0.001) implying that relative to the Chinese participants, the American participant’ feelings of depression were more sensitive to closeness. The three-way interaction between closeness, fulfillment, and country was also significant (*B* ± *S.E*.= 4.002 ± 1.29, 95% CI = [1.50, 6.51], *b* = 0.806, *t* = 3.113, *p* < .01) suggesting that the moderating role of closeness was opposite between the two groups (Fig. 2C). Chinese participants reported greater feelings of depression when failing to fulfill expectations of those closer to them while American participants felt the least depression in the same instance.

### Behavior Change

Across countries, degree of fulfillment was positively associated with the tendency to alter their behavior in order to completely fulfill the expectation (*B* ± *S.E*.= 0.396 ± 0.068, 95% CI = [0.262, 0.527], *b* = 0.359, *t* = 5.837, *p* < .001). There was no main effect of country (*t* = -0.419; *p* = .68). There was a significant interaction between country and fulfillment, such that Chinese participants’ tendency to change their behavior was more sensitive to fulfillment (*B* ± *S.E*.= -0.253 ± 0.116, 95% CI = [-0.477, -0.027], *b* = -0.28, *t* = -2.19; *p* = .03). More specifically, the degree to which Chinese participants were willing to alter their behavior was more closely correlated with the degree to which they had fulfilled the expectation than for the American participants. 

**Fig. 3:**
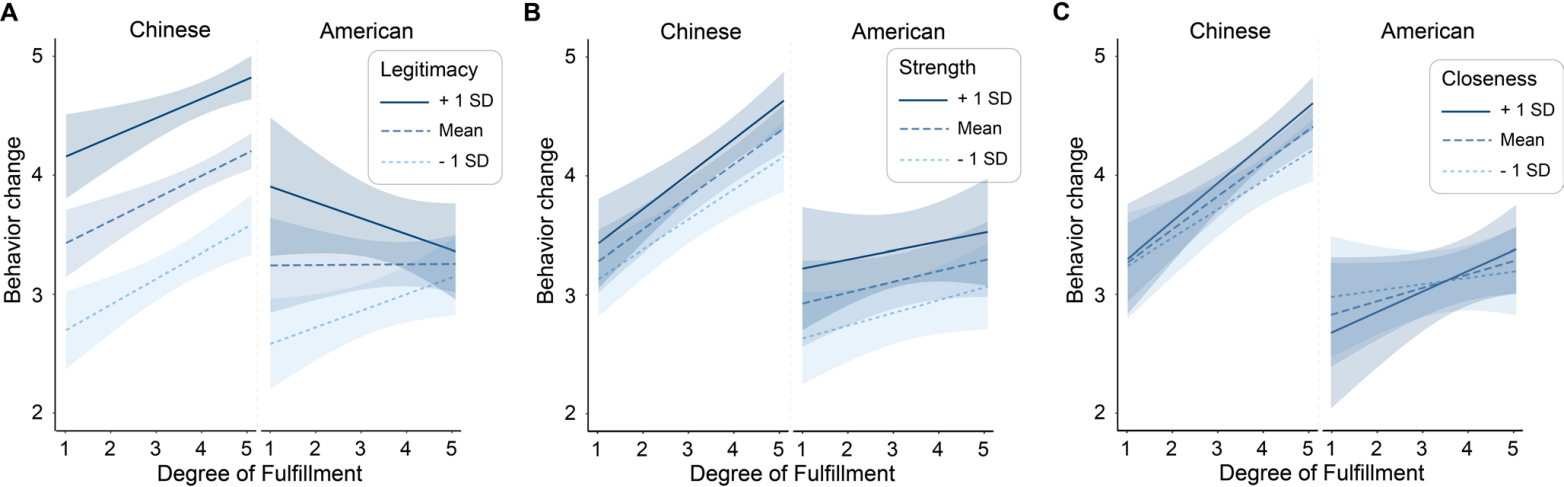
Effects of expectation fulfillment on behavior change, modulated by situational factors - legitimacy of expectation (**A**), strength of expectation (**B**), and closeness to the source of expectation (**C**)

Across countries, behavior change was positively associated with perceived legitimacy (*B* ± *S.E*.= 0.759 ± 0.135, 95% CI = [0.49, 1.02], *b* = 0.668, *t* = 5.604; *p* < .001) such that more legitimate expectations were associated with an increased tendency to alter behavior in order to fulfill the expectation (Fig. 3A). We found no influence of strength or closeness on behavior change (Fig. 3B C).

## Discussion

Study 1 used a daily diary design to examine how failing to fulfill perceived social expectations relates to expectation recipients’ emotions and behavioral tendencies, and whether situational factors and cultural context moderate these links. As predicted, lower fulfillment (i.e., larger expectation violations) was associated with higher negative affect, especially guilt and depression, consistent with prior work linking expectation discrepancies to distress (Agliata & Renk, 2009). Contrary to prediction, intentions to change behavior increased when participants were closer to fulfilling expectations. This “recency effect” may reflect attainability: when full fulfillment seems within reach, people may be more motivated to adjust. Future work should disentangle whether behavioral change is driven primarily by guilt, perceived attainability, or both.

Cultural moderation was mixed. Country differences were not reliable for guilt but emerged for depression: U.S. participants reported higher depression overall, particularly at low fulfillment. Chinese participants showed a tendency toward lower guilt and greater willingness to change behavior to meet expectations, which may buffer negative consequences and aligns with evidence that East Asian socialization emphasizes conformity and meeting expectations (Bornstein, 2005; Choi et al., 2013; Fuligni, 2007; Naumann et al., 2012).

Situational factors mattered: stronger and more legitimate expectations elicited greater negative affect, and closeness showed a cultural divergence—U.S. participants felt less negative when violating close others’ expectations, whereas Chinese participants felt more negative in close relationships—consistent with cultural differences in interdependence and relational obligation (Markus & Kitayama, 1991; Mesquita, 2022). Exploratory individual-level cultural-orientation analyses (Eom et al., 2016; Triandis, 1989) suggested convergent patterns but warrant cautious interpretation (Supplementary Materials: 2.1 Intake Data Analysis).

## Study 2

Study 1 showed that situational appraisals (e.g., strength, legitimacy, closeness) moderated how fulfillment related to negative affect and behavior change, underscoring that recipients’ interpretations shape emotional responses. This raises a key question: how accurate are these self-evaluations? Because people are embedded in the relationships that generate expectations, their judgments may reflect bias, emotional involvement, or motivated reasoning (Burgoon & Hale, 1988; Higgins, 1987), potentially amplified in close relationships and varying across cultures (Markus & Kitayama, 1991; J. G. Miller & Bersoff, 1992).

### Method

#### Study Overview

Study 2 tests whether recipients’ evaluations systematically deviate from a disinterested benchmark by obtaining third-party ratings of the same expectations. We treat third-party judgments not as “objective truth,” but as a neutral reference point to quantify the direction and magnitude of bias and examine its affective consequences. We preregistered hypotheses and analyses for both samples (US: https://aspredicted.org/FW9_H1D; Chinese: https://aspredicted.org/Z9W_HZ1).

In Study 1, we found that situational factors, to different degrees, moderated the effect of fulfillment on negative affect and behavior change in both the American and Chinese participants. These findings highlight that expectation receivers’ interpretations matter greatly for understanding their emotional responses. However, this also raises an important question: *How accurate are these self-evaluations?* Since individuals are embedded within the relationships that give rise to expectations, their judgments may reflect personal biases, emotional involvement, or motivated reasoning (Burgoon & Hale, 1988; Higgins, 1987). In other words, people may not simply report the expectations placed upon them—they may also interpret, amplify, or downplay these expectations in systematic ways.

This issue is particularly relevant in close relationships, where expectations from parents, partners, and family members carry unique emotional weight. A substantial literature demonstrates that expectation discrepancies in close relationships—especially with parents—are strongly associated with guilt, distress, and reduced well-being (Agliata & Renk, 2009; Saw et al., 2013). Research on parental expectations specifically shows that failing to meet expectations from close others can undermine autonomy, generate self-blame, and heighten emotional distress, particularly when expectations have been internalized (Shen et al., 2014). Moreover, these dynamics can differ cross-culturally: individuals from collectivistic contexts often place greater moral weight on meeting the expectations of close others (Miller & Bersoff, 1992; Oishi & Sullivan, 2005), whereas those from individualistic cultures may interpret the same expectations through a lens of autonomy and psychological independence (Markus & Kitayama, 1991). These cultural differences suggest that self-evaluations of expectation strength, legitimacy, and closeness may themselves be culturally biased.

Study 2 was therefore designed to determine whether the subjective evaluations observed in Study 1 reflect systematic cognitive or cultural biases,. By obtaining third-party ratings of the same expectations reported by Study 1 participants, we created a neutral comparison point—not to identify a single “correct” value, but to examine the direction and magnitude of participants’ deviations from a disinterested evaluator. Third-party ratings provide methodological leverage for testing whether expectation recipients interpret their social environment in ways that amplify emotional burden, consistent with theories of motivated social cognition and interpersonal obligation (Baumeister et al., 1994; Theriault et al., 2021).

Establishing these biases is theoretically important for several reasons. First, it clarifies whether emotional distress in response to unmet expectations stems primarily from the expectations themselves or from how individuals subjectively construe them—echoing findings in the parental expectations literature that interpretations are often more predictive of affect than objective expectation levels (Agliata & Renk, 2009; Shen et al., 2014). Second, identifying cultural differences in interpretive bias helps explain why expectation violations elicit stronger negative emotions in some cultural contexts than others (Oishi & Sullivan, 2005; Mesquita, 2022). Finally, understanding these interpretive tendencies has implications for mental health: individuals who chronically overestimate the strength or legitimacy of others’ expectations may inadvertently heighten their own vulnerability to guilt and depression.

(https://aspredicted.org/Z9W_HZ1) https://aspredicted.org/FW9_H1D)

#### Participants

American (*N* = 54) and Chinese (*N* = 177) undergraduate students were recruited through Prolific or university-based recruitment programs. Of these, 30 participants (14 American and 16 Chinese participants) were excluded from the final analysis due to failing the attention check questions. The final sample consisted of 201 participants (American: *N* = 40, 22 male participants, *M*_age_ = 26.51, *SD*_age_ = 6.54; Chinese: *N* = 161, 33 male participants, *M*_age_ = 18.11, *SD*_age_ = 0.81) who rated a total of 958 expectations (375 American and 583 Chinese expectations). The American participants only read the expectations reported by the original American participants written in English, and the Chinese participants only read the expectations reported by the original Chinese participants written in Chinese.

#### Procedure

Study 1 required participants to complete an open-ended response where they described the expectation they were reporting on. These open-ended responses were extracted, cleaned for any potential typos and identifiable information, edited to include the source of the expectation if it did not already include one, and then displayed to third-party participants. Each participant of Study 2 rated about 30 of these expectations, from a detached, third-person perspective, on how strong and legitimate the expectations seemed to them, as well as the perceived closeness of the two people interacting within the expectation. (We found no notable effects of closeness in our analyses and therefore will not report them below.) Each expectation was rated by at least three new participants from the same country as the original participants (American or Chinese). The raters’ scores of each expectation were then averaged to give us one new score to compare to the original ratings.

### Analysis Plans

We preregistered our analysis plan and detailed a linear mixed model that included the strength and legitimacy scores as the outcome variables, the type of rating (original vs. third-party), country, as well as the interaction between the two as the primary predictor variables. We also planned to include both the original participant ID and the expectation ID as random intercepts. However, we found that this model was overfitting our data and therefore simplified it by removing the expectation ID random factor.

For our hypothesis-driven analysis, we examined whether the type of rating had a main effect on the outcome variables (i.e., strength and legitimacy of expectations). As a robustness check, in a separate set of models, we added original participants’ demographic information as control variables.

If evaluation perspective influences perceived strength and legitimacy of social expectations, to what extent would these biases amplify the negative emotions participants experienced compared to a more neutral, third-party perspective? We applied the legitimacy and strength models from Study 1, integrating the newly obtained legitimacy and strength ratings, to predict the levels of guilt, depression, and behavior change that the original participants would have reported if they had evaluated the expectations from a third-party perspective. We used the beta values from the legitimacy and strength models which included legitimacy/strength, fulfillment, country, and the interactions between the three as the primary predictor variables. The model also included demographic variables as covariates of no interest, as well as the original participant ID as a random factor. We then used these model-based values in two ways. We first computed a percentage difference between the model-based and original ratings of negative affects and behavior change. For example, the percentage difference of guilt is defined as:

$$Difference\%=(guilt{\prime}-guilt)/guilt$$

where *guilt’* is the model-based guilt and *guilt* is the guilt reported by the original participants. This percentage will show us the extent to which the original participants “over-estimated” (if the percentage is positive) or “underestimated” (if the percentage is negative) their feelings. We also formally evaluated whether the model-based ratings and original ratings differ significantly. To do this, we used a linear mixed-effects model in which the guilt, depression, and behavior change values were the primary outcome variables and the value type (i.e., model-based vs. original) was the primary predictor variable.

## Results

### Descriptive Analysis

Since this follow-up study was a comparison with the ratings provided by two groups of participants, we first ensured that the samples from Studies 1 and 2 were not significantly different. We performed a χ^2^ test of proportions and found that the gender distribution between the participant groups were comparable (χ^2^ (1, *N* = 536) = 1.85, *p* = .17). We found through an independent samples *t*-test that there was no significant difference in the age of the two samples (Study 1: *M*_age_ = 20.5, *SD*_age_ = 3.5; Study 2: *M*_age_ = 22.31, *SD*_age_ = 3.68; *t*(311.52) = 0.393, *p* = .69).

We also assessed the inter-rater reliability of the ratings of reasonableness (α = 0.74), warrantedness (α = 0.7), justifiability (α = 0.73), and strength (α = 0.42) across each of the expectations, and found that all but the consensus on strength of expectations was high. The low consensus of strength was expected, as strength of expectations is operationalized as the mode of expression which is more difficult to capture in the verbal description of the expectation itself. Therefore, the results pertaining to strength in the following analyses should be interpreted cautiously.

### Hypothesis-Driven Analysis: The Effect of Perspective on Perceived Strength and Legitimacy of Social Expectations

We first assessed the ratings of strength and found that the ratings significantly differed as a function of perspective (*B* ± *S.E.* = 0.312 ± 0.044, 95% CI = [0.226, 0.399], *b* = 0.193, *t* = 7.102, *p* < .001), such that the original participants rated the expectations as stronger than the third-party observers. We also observed a main effect of country (*B* ± *S.E*. = -0.186 ± 0.059, 95% CI = [-0.30, -0.07], b = -0.111, *t* = -3.137, *p* = .002) such that American participants generally reported expectations as weaker than Chinese participants, mirroring our original findings. We found no interaction effect of country and perspective.

Similarly, we found that legitimacy also varied by perspective (B ± S.E. = 0.235 ± 0.052, 95% CI = [0.134, 0.336], b = 0.118, *t* = 4.555, *p* < .001), such that the original participants rated the expectations as more legitimate than third-party observers. We again found a significant main effect of country (*B* ± *S.E.* = -0.498 ± 0.075, 95% CI = [-0.645, -0.352], *b* = -0.245, *t* = -6.653, *p* < .001) such that American participants rated expectations as less legitimate than Chinese participants. We again found no interaction between country and perspective. We note here that the effect sizes for both models were small, indicating that although there was a significant difference, the difference itself was not large.

### Robustness Check

We next ran the same model described above but included the original participants’ demographics information (socioeconomic status, sex, and age) to check the robustness of the findings from the hypothesis-driven models. Looking first at the model for strength, we found that the effect continues to be significant (*B* ± *S.E*. = 0.31 ± 0.44, 95% CI = [0.23, 0.40], *b* = 0.193, *t* = 7.114, *p* < .001). We also continue to observe the main effect of country (*B* ± *S.E*. = -0.197 ± 0.622, 95% CI = [-0.319, -0.076], *b* = -0.118, *t* = -3.175, *p* = .002). Our legitimacy models also revealed a sustained main effect of legitimacy (*B* ± *S.E*. = 0.234 ± 0.516, 95% CI = [0.133, 0.335], *b* = 0.118, *t* = 4.536, *p* < .001) and country (*B* ± *S.E.* = -0.508 ± 0.783, 95% CI = [-0.661, -0.356], *b* = -0.25, *t* = -6.493, *p* < .001), suggesting that these are fairly robust effects across groups.

### Exploratory Analysis: Overview

We conducted two types of analyses to explore our data further. The first served as a robustness test of the modulatory effects of perceived legitimacy and strength of social expectations on the relationship between expectation fulfillment and negative affects. We assessed whether the new ratings of legitimacy and strength would moderate the effects of failing to fulfill social expectations on the original ratings of guilt, depression, and behavior change in a comparable manner as the original ratings of perceived legitimacy and strength. In the second analysis, we used the new ratings of legitimacy and the original legitimacy models that included degree of fulfillment, legitimacy, country, their interactions, as well as demographic variables to calculate a model-based guilt, depression, and behavior change. We then used these model-based measures to understand the influence of perspective on the primary dependent variables. The following sections will detail these analyses further. For this second analysis, we focused on perceived legitimacy because it was the only situational factor that consistently modulated the relationship between expectation fulfillment and negative affects in Study 1, and because the inter-rater reliability of the third-party evaluation of legitimacy (but not strength) was high.

### Exploratory Analysis: Moderation Analysis with the Third-Party Evaluation of Situational Factors of Social Expectations

We ran linear mixed models with the original ratings of the primary dependent variables (guilt, depression, and behavior change) as the outcome variables. The original degree of fulfillment, and the new strength or legitimacy ratings (as the moderation factor), country, as well as interactions between the three were the predictors. We also included the original participant IDs as a random factor.

#### Guilt

We found a main effect of the new legitimacy ratings in predicting guilt (*B* ± *S.E*. = 11.373 ± 3.401, 95% CI = [4.716, 18.016], *b* = 0.35, *t* = 3.344, *p* < .001), such that more legitimate ratings were associated with greater guilt. We also observed an interaction effect between legitimacy and fulfillment (*B* ± *S.E*. = -2.056 ± 0.974, 95% CI = [-3.96, -0.15], *b* = -0.404, *t* = -2.111, *p* = .035), such that the effects of legitimacy on guilt were stronger at lower levels of fulfillment, replicating the patterns observed in Study 1 when original legitimacy ratings were used as the modulatory factor. However, similar to the main findings, we found no effects of strength.

#### Depression

We found no main effect of legitimacy but found a significant legitimacy-by-country interaction (*B* ± *S.E.* = 9.344 ± 4.043, 95% CI = [1.431,17.242], *b* = 0.608, *t* = 2.311, *p* = .02), such that greater depression was associated with expectations that were higher in legitimacy, and this especially true for American participants. Again, this pattern was similar to the one found using the original legitimacy ratings. As before, we found no significant effects of strength on feelings of depression, similar to patterns based on the original strength ratings.

#### Behavior Change

Similar to the original findings, we found that behavior change was positively associated with legitimacy (*B* ± *S.E*. = 0.698 ± 0.161, 95% CI = [0.384, 1.012], *b* = 0.547, *t* = 4.345, *p* < .001) such that more legitimate expectations were more likely to elicit a greater motivation to change behavior in order to fulfill the expectation. We found no main effect of country or interaction between country and legitimacy (*t* < |1.8|, *p* > .07). Similar to the original findings, we found no effects of strength.

### Exploratory Analysis: Comparing the Model-Based and Original Negative Affects and Behavior Change

#### Guilt

Our model-based guilt revealed that the overestimation of legitimacy resulted in the original participants experiencing 10% more guilt than the third-party raters would have experienced, while an overestimation of strength resulted in 12% more guilt. This was reflected in the mixed models, which revealed a main effect of type for legitimacy (*B* ± *S.E*. = 3.314 ± 1.246, 95% CI = [0.87, 5.76], *b* = 0.073, *t* = 2.659, *p* = .008) on model-based guilt such that, on average, the original rating of guilt was greater than the model-based one. We found a similar main effect of strength (*B* ± *S.E*. = 3.967 ± 1.247, 95% CI = [1.523, 6.413], *b* = 0.088, *t* = 3.182, *p* = .002) on model-based guilt such that the original rating of guilt was greater than the model-based one.

#### Depression

In contrast to guilt, overestimation of legitimacy and strength resulted in only a 5% and 8% increase in depression in the original participants. We found a main effect of type in only the strength model-based depression (*B* ± *S.E*. = 1.922 ± 0.912, 95% CI = [0.13, 3.71], *b* = 0.04, *t* = 2.109, *p* = .035) but not in legitimacy model-based depression (*t* = 1.296; *p* = .195), suggesting that this overestimation may be specific to the negative affect of guilt and not depression.

#### Behavior change

The difference between model-based and original behavior change was less than 1% in both legitimacy and strength models. This was reflected in the mixed models in which we did not find a main effect of type (*t*s < 1.74; *p*s > 0.082). However, we found a main effect of country in both the legitimacy (*B* ± *S.E.* = -0.69 ± 0.15, 95% CI = [-0.99, -0.39], *b* = -0.209, *t* = -4.50, *p* < .001) and strength (*B* ± *S.E.* = -0.717 ± 0.159, 95% CI = [-1.001, -0.431], *b* = -0.228, *t* = -4.923, *p* < .001) model-based behavior change, such that American participants generally reported lower likelihoods of altering behavior. More interestingly, we also found an interaction effect between country and type (legitimacy model: *B* ± *S.E*. = -1.032 ± 0.173, 95% CI = [-1.37, -0.69], *b* = -0.229, *t* = -5.98, *p* < .001; strength model: *B* ± *S.E*. = -1.002 ± 0.159, 95% CI = [-1.313, -0.692], *b* = -0.233, *t* = -6.322, *p* < .001) implying that behavior change was influenced by perspective but only for American participants, while remaining consistent for Chinese participants. This continues a theme from the pilot studies in that the variable of behavior change responds to situational factors somewhat differently than affect and is particularly sensitive to culture.

## Discussion

Study 2 examined whether expectation recipients’ appraisals differ from detached observers and whether such differences matter for affect. We found reliable perspective effects: original participants rated the same expectations as more legitimate and stronger than third-party raters. Substituting third-party evaluations into the Study 1 models reduced predicted guilt (and, modestly, depression), indicating that recipients’ higher self-ascribed legitimacy/strength can inflate violation-induced negative affect. The participant sample (U.S. vs. China) did not moderate perspective effects.

Why does perspective seem to have a stronger effect on guilt than depression? One plausible account concerns appraisal match. Guilt is a self-conscious, event-related emotion tightly coupled to obligation/responsibility appraisals; inflating legitimacy increases perceived “oughtness,” directly amplifying guilt (Haidt, 2003; Tangney et al., 2007; Yu et al., 2024). The depression measure reflects broader, mood-like symptoms over a longer window. Therefore, a single episode’s re-appraisal will exert a smaller effect.

Some limitations of this study are worth noting. First, the third-party judgments were based on short textual descriptions; future studies should include partner reports or dyadic data (i.e., both expectation holder and expectation recipient). Second, the inter-rater reliability for strength was quite low, likely reflecting its reliance on tone/mode of expression that is hard to infer from text.

## General Discussion

Prior research on social expectations has largely emphasized the expectation holder, asking how people react when others fall short (Burgoon & Hale, 1988; McNulty & Karney, 2004; Mellers et al., 1997; Schultz, 1997, 1998). The present work extends this framework by focusing on the expectation recipient—how emotions arise when individuals believe they have violated others’ expectations. Study 1 replicated and extended these findings using a five-day daily diary design that captured expectations in everyday life and modeled fulfillment as a continuum rather than a categorical violation. Consistent with prediction-error accounts, lower fulfillment was linked to greater guilt and depression (Kishida & Sands, 2021; Schultz, 2015; Villano et al., 2020, 2023). We also examined situational “gating” factors that may signal the importance of an expectation. Legitimacy reliably amplified negative affect, consistent with the idea that people weigh expectations they perceive as reasonable or meaningful (Burgoon, 1993; Earp et al., 2021). Strength showed a more mixed pattern: although stronger expectations related to greater guilt and depression in recall-based reports, they were less predictive of behavior change in daily life, suggesting that recognizing a strong expectation does not always translate into action when feasibility and competing demands constrain behavior.

Exploratory cultural analyses suggested both commonalities and differences. In both the U.S. and China, unmet expectations predicted guilt and depression, but moderators differed: U.S. participants were more sensitive to legitimacy, whereas Chinese participants were more sensitive to relational closeness. Notably, closeness showed opposite effects across countries—U.S. participants reported less negative emotion when violating close others’ expectations, whereas Chinese participants reported more guilt and distress—consistent with cultural differences in autonomy versus relational obligation (Kim et al., 2006; Markus & Kitayama, 1991).

Study 2 addressed whether recipients’ situational evaluations are biased by comparing their ratings to third-party judgments. Recipients rated expectations as more legitimate and stronger overall, and this pattern appeared similar across countries. Importantly, greater “overestimation” predicted stronger guilt (but not depression), suggesting guilt may be especially sensitive to perspective-taking. This has potential intervention implications: helping people evaluate expectations more distantly may reduce excessive guilt and associated burden.

This work has several limitations. First, we focused on the recipient side of expectation violation; as a result, we cannot capture the full, dynamic interplay between expectation holders and recipients, nor can we reveal how emotions circulate within the dyad to escalate or repair expectation violations. Addressing this will require dyadic, longitudinal designs that measure both partners’ expectations, emotions, and behaviors over time. Second, we examined only failures to fulfill expectations, leaving untested the affective and motivational profiles of exceeding expectations. These cases are theoretically important and likely more complex—exceedance does not always yield pleasant outcomes and may prompt discomfort or avoidance depending on relationship orientation (e.g., communal vs. exchange; (Clark & Mils, 1993). Future research is needed to examine the full range of violating, meeting, and exceeding expectations, preferably in a dyadic context. Third, we treated situational appraisals (e.g., legitimacy, strength) as antecedents of emotion and emphasized the correlational nature of our analyses; however, emotions may also shape these appraisals (e.g., depressive states could inflate perceived strength and/or legitimacy).

In sum, this research extends existing theories on social expectations to expectation violations from the recipient’s perspective. We demonstrate that across both China and the U.S., failing to fulfill social expectations elicit negative emotions, with legitimacy and strength of the expectation serving as key modulators of this effect. Moreover, our findings suggest that expectation recipients overestimate the legitimacy (and to a lesser degree, strength) of the expectation, thereby amplifying their negative social emotions, especially guilt. By illuminating how situational appraisals and perspective shape emotion, this research has implications for designing intervention—in communication training, clinical reappraisal, and relationship education—and sets the stage for dyadic, longitudinal tests of how partners co-construct expectations and emotions over time.

## Supplementary Information

Below is the link to the electronic supplementary material.

Supplementary Material 1.

## Additional Information

### Availability of Data and Material

The experimental data and the analysis codes that support the findings of this study are available on OSF: https://osf.io/js83k/
