# Supplementary material for: Failing Others’ Expectations: Negative Emotions and Behavior Change in Daily Life
Source: Affect Sci. 2026 Apr 23;7(2):319–32. doi: 10.1007/s42761-026-00365-w (PMC13269612; doi:10.1007/s42761-026-00365-w)
Supplement: Supplementary file 1 — Supplementary Material 1. [file 42761_2026_365_MOESM1_ESM.pdf]

Supplementary Materials for

Failing Others' Expectations:

Negative Emotions and Behavior Change in Daily Life

Table of Contents

1. Pilot Study ***1***

1.1 Methods **1**

1.1.1 Participants **1**

1.1.2 Analysis **2**

1.2 Results 2

1.2.1 Cohort 1: Legitimacy manipulation 2

1.2.2 Cohort 2: Strength manipulation 3

2. Supplementary Results of Study 2 .... ***4***

2.1 Intake Data Analysis **5**

2.2 Other dependent measures from daily diary **10**

2.3 Bayesian Robustness Check **11**

2.4. Situational factors as a function of country **18**

2.5 Mediation analysis **18**

2.6 Descriptive Statistics **20**

3. Supplementary Figures ***23***

1. Pilot Study

The pilot study followed the same manipulation of legitimacy (Cohort 1) and strength (Cohort 2) described in Study 1 in the main text. Similarly, legitimacy was measured as a composite score of perceived justifiability, warrantedness, and reasonableness of the social expectation (Cronbach αs > 0.89).

1.1 Methods

1.1.1 Participants

Cohort 1: Legitimacy manipulation. We recruited American undergraduate students from Prolific and university-based recruitment platforms (N = 85). Twelve participants were excluded from the analyses due to failure in attention check questions, leaving a final sample of 73 (47 female participants, M_{age} = 19.4, SD_{age} = 2.7).

Cohort 2: Strength manipulation. We recruited American undergraduate students from Prolific and university-based recruitment platforms (N = 88). Eleven participants failed the attention check questions and were excluded from the analyses, leaving a final sample size of 77 (58 women, M_{age} = 18.9, SD_{age} = 1.1).

1.1.2 Analysis

Model specification. We analyzed the data using linear mixed effects models with the primary dependent variables of guilt, stress, and behavior change (likelihood to apologize, avoid, and compensate their hypothetical partner) as the outcome variables and legitimacy or strength condition as the predictor variable. We included the demographic variables of gender, age, number of siblings, and socioeconomic status as fixed effect control variables, and subject ID and scenario version as random intercepts. We found that our specified model was overfitting the data. Upon closer inspection, the variance between scenario versions was negligible suggesting that using it as a random factor was redundant and therefore. We therefore dropped the scenario version as a random factor. The results reported below were the findings from this simplified model.

1.2 Results

1.2.1 Cohort 1: Legitimacy manipulation

Manipulation check. We ran the specified mixed effects model and found that participants reported the high legitimacy expectations as significantly more legitimate than the low legitimacy expectations (B ± S.E.= 1.003 ± 0.172, 95% CI = [0.656, 1.334], b = 0.397, $t = 5.829$, $p < .001$), suggesting a successful manipulation of legitimacy.

Hypothesis-driven tests. We found that the legitimacy condition had no effect on guilt, stress, or the behavior change variables (ts < 1.971, ps > 0.52).

Exploratory tests. We next ran the same simplified models using perceived legitimacy and expectation condition as the primary explanatory variables. We found that legitimacy was

significantly associated with guilt (B ± S.E.= 5.39 ± 1.74, 95% CI = [1.641, 8.769], b = 0.273, t = 3.094, p = .002) but not stress (t = 0.384, p = .701). We also found that legitimacy was positively associated with the tendency to compensate (B ± S.E.= 0.584 ± 0.128, 95% CI = [0.338, 0.834], b = 0.382, t = 4.548, p < .001) and apologize (B ± S.E.= 0.23 ± 0.10, 95% CI = [0.032, 0.427], b = 0.203, t = 2.237, p = .027) but not avoid (t < |1.035|, p > .302) their hypothetical partner.

1.2.2 Cohort 2: Strength manipulation

Manipulation check. We ran the specified mixed effects model and found that participants reported high strength expectations as significantly stronger that the low strength expectations (B ± S.E.= 0.818 ± 0.224, 95% CI = [0.386, 1.251], b = 0.283, t = 3.657, p < .001), suggesting a successful manipulation of strength.

Hypothesis-driven tests. We found that participants' self-reported guilt, stress, and behavior did not vary as a function of strength condition (all ts < 1.749, ps > .084).

Exploratory tests. We estimated a separate set of linear mixed effects models similar to the one described above but instead used perceived strength as the primary predictor variable. We found that guilt (B ± S.E.= 2.939 ± 1.239, 95% CI = [0.57, 5.43], b = 0.196, t = 2.373, p = .019) but not stress (t = 1.386, p = .169) was a significant predictor, such that failing to fulfill stronger expectations was associated with greater feelings of guilt. We also found positive associations between perceived strength and the likelihood to apologize (B ± S.E.= 0.359 ± 0.072, 95% CI = [0.22, 0.50], b = 0.394, t = 5.01, p < .001) and compensate (B ± S.E.= 0.215 ± 0.107, 95% CI =

[0.01, 0.43], b = 0.151, $t = 2.018$, $p = .046$), but not avoid ($t = |1.303|$, $p = .195$) their hypothetical partners.

2. **Supplementary Results of Study 2**

Below, we report the additional analyses from Study 2. First, we conducted the same set of analyses that we did for the daily diary task on the intake data during which participants were asked to report on an expectation they had experienced in the previous 2 weeks. The intake and daily diary analyses were separated due to the nature of the task (recall versus same-day experiences).

Second, we reported the results of other, exploratory variables in the daily diary survey.

Third, as mentioned in the main text, some of our dependent variables were not normally distributed. We therefore replicated the key analyses of the daily diary data using Bayesian regression, which is less susceptible to the distribution of data (Smith, Spiegelhalter, & Thomas, 1995). Overall, most of the effects we reported in the main text based on linear mixed effects models were replicated using Bayesian regression, indicating robustness of our results.

Fourth, we examined whether and how our key situational factors (i.e., legitimacy and strength of the reported expectations, and closeness to the expectation holder) vary systematically between our Chinese and American samples.

Fifth, we ran a mediation analysis based on the daily diary data to explore the mediating role of guilt in the relationship between fulfillment of social expectations and behavior change.

Sixth, we ran some analysis to estimate the achieved power of the daily diary study.

Finally, we reported some descriptive statistics of the daily diary data of Study 1.

2.1 Intake Data Analysis

The following findings are from the analysis of the intake session in which participants recalled an expectation they had experienced during the two weeks prior to taking the survey. They reported on the same variables as mentioned for the daily diary section. Additionally, we also measured person-level individualistic and collectivistic tendencies (Eom et al., 2016), and reported some of the moderating effects of these cultural orientations.

2.1.1 Guilt.

Guilt was negatively associated with degree of fulfillment across countries (B ± S.E. = -9.627 ± 1.625, 95% CI = [-12.891, -6.401], b = -0.42, t = -5.923, p < .001) such that participants felt less guilt at greater levels of fulfillment. However, this effect was not moderated by country.

Similar to the daily diary data, we found a main effect of perceived legitimacy (B ± S.E. = 11.66 ± 4.25, 95% CI = [3.436, 19.882], b = 0.403, t = 2.743, p = .006), such that more legitimate expectations were more likely to incite greater feelings of guilt. We also observed an interaction effect between legitimacy and degree of fulfillment of the expectation (B ± S.E. = -3.095 ± 1.408, 95% CI = [-5.818, -0.372], b = -0.694, t = -2.199, p = .028) implying that legitimacy had a greater effect on guilt when the expectation was farther away from fulfillment.

We found no main effect of strength, but found a strength-by-country interaction (B ± S.E. = 15.004 ± 7.066, 95% CI = [1.332, 28.676], b = 0.998, t = 2.123, p = .034) such that American participants were more sensitive to the effects of strength of social expectations. We also observed a marginal three-way interaction between strength, degree of fulfillment of the

expectation, and country (B ± S.E. = -3.485 ± 2.002, 95% CI = [-7.358, 0.388], b = -0.951, t = -1.741, p = .082).

We found no significant effects pertaining to closeness, contrary to the daily diary data and the Bayesian robustness check, suggesting that the intake data alone might be underpowered to detect the effect.

We found a main effect of individualism on guilt (B ± S.E. = 12.646 ± 5.721, 95% CI = [1.574, 23.712], b = 0.379, t = 2.21, p = .028) such that more individualistic individuals were more likely to experience greater feelings of guilt. We also found an interaction between individualism and country (B ± S.E. = -24.469 ± 8.029, 95% CI = [-39.998, -8.927], b = -0.53, t = -3.047, p = .002) such that more individualistic American participants felt less guilt, while the opposite was true for the Chinese participants. These effects were then observed in a significant three-way interaction between individualism, country, and fulfillment (B ± S.E. = 5.382 ± 2.271, 95% CI = [0.984, 9.773], b = 0.448, t =2.37, p = .018) in which individualism had the opposite moderating effect for participants from the two countries. At lower levels of fulfillment, more individualistic American participants felt less guilt while more individualistic Chinese participants felt greater guilt. We observed no effects of family achievement guilt.

2.1.2 Depression.

Feelings of depression were negatively associated with degree of fulfillment of the expectation B ± S.E. = -6.866 ±1.558, 95% CI = [-9.902, -3.827], b = -0.316, t = -4.407, $p < .001$) such that participants reported feeling greater feelings of depression when they were farther away from fulfilling a social expectation. However, we found no main or interaction effects of country.

Similar to the daily diary data analysis, we find no direct effects of perceived legitimacy on depression but observe an interaction effect between legitimacy and country (B ± S.E. = 14.69 ± 6.57, 95% CI = [1.97, 27.398], b = 0.972, $t = 2.235$, $p = .026$) such that American participants were more sensitive to the legitimacy of expectations. Although not significant, we see a marginal three-way interaction between legitimacy, fulfillment, and country (B ± S.E. = -3.242 ± 1.954, 95% CI = [-7.026, 0.543], b = -0.921, $t = -1.659$, $p = .097$). We find a rather strong effect in both the daily diary data and the Bayesian robustness check, suggesting that the intake data alone is underpowered to detect this effect.

We find no direct effects of perceived strength on depression but find an interaction effect between strength and country (B ± S.E. = 19.797 ± 7.052, 95% CI = [6.117, 33.495], b = 1.369, $t = 2.807$, $p = .005$) such that American participants were more sensitive to the effects of strength. We found other effects of strength and no effects of closeness.

Similar to guilt, we found that individualism was positively associated with feelings of depression (B ± S.E. = 14.185 ± 5.809, 95% CI = [2.944, 24.427], b = 0.442, $t = 2.442$, $p = .015$) such that participants who were more individualistic were more likely to report greater depression. This effect, however, was moderated by country (B ± S.E. = -21.38 ± 8.18, 95% CI = [-37.212, -5.545], b = -0.481, $t = -2.613$, $p = .009$) such that American participants who were lower in individualism were more likely to report greater depression. There was also a marginal three-way interaction between individualism, fulfillment, and country (B ± S.E. = 4.512 ± 2.309, 95% CI = [0.043, 8.983], b = 0.39, $t = 1.954$, $p = .05$) such that the interaction between individualism and country was reversed for Chinese participants, who were also generally more sensitive to individualism at lower levels of fulfillment.

2.1.3 Behavior Change.

We found a main effect of degree of fulfillment of the expectation on behavior change (B ± S.E. = 0.463 ± 0.091, 95% CI = [0.286, 0.639], b = -0.921, t = 5.093, p < .001) such that participants reported being more likely to alter behavior in order to fulfill the expectation the closer they were to fulfilling the expectation. We also found that this effect was moderated by country (B ± S.E. = -0.306 ± 0.126, 95% CI = [-0.549, -0.063], b = -0.399, t = -2.437, p = .015) such that American participants reported being less likely to alter their behavior in order to fulfill expectations, in contrast to their Chinese counterparts.

We found main effects for legitimacy (B ± S.E. = 0.77 ± 0.18, 95% CI = [0.426, 1.119], b = 0.672, t = 4.293, p < .001), strength (B ± S.E. = 0.521 ± 0.249, 95% CI = [0.041, 1.001], b = 0.424, t = 2.091, p = .038), and closeness (B ± S.E. = 0.565 ± 0.209, 95% CI = [0.163, 0.966], b = -0.921, t = 2.705, p = .007). More legitimate, stronger, and expectations from closer others were positively associated with a greater likelihood of altering behavior in order to fulfill the expectation. We found no other interaction effects for the situational factors.

We find a marginal main effect of family achievement guilt on behavior change (B ± S.E. = 0.339 ± 0.204, 95% CI = [-0.053, 0.732], b = 0.335, t = 1.664, p = .097) such that participants higher on family achievement guilt were more likely to alter their behavior in order to fulfill an expectation. We find no other effects of family achievement guilt, and also observed no impact of individualism on behavior change.

2.1.4 Stress.

We found that degree of fulfillment of the expectation was negatively associated with stress (B ± S.E. = -8.846 ± 1.756, 95% CI = [-12.264, -5.427], b = -0.361, $t = -5.033$, $p < .001$) such that lower levels of fulfillment were associated with greater levels of stress.

We found a main effect of strength (B ± S.E. = 13.705 ± 5.815, 95% CI = [2.434, 24.997], b = 0.422, $t = 2.357$, $p = .019$), such that stronger expectations were more strongly associated with feelings of stress. We found no effects of perceived legitimacy or closeness to the source of the expectation.

We found no main effect of individualism, but similar to other affect, found an interaction between individualism and country (B ± S.E. = -22.105 ± 9.155, 95% CI = [-39.82, -4.39], b = -0.44, $t = -2.414$, $p = .016$), such that more individualistic American participants were less likely to experience stress while the opposite was true for Chinese participants. We found no effects of family achievement guilt.

2.1.5 Self-esteem.

We found no effect of fulfillment but observed a main effect of country on self-esteem (B ± S.E. = -25.462 ± 7.546, 95% CI = [-40.14, -10.79], b = -0.45, $t = -3.374$, $p < .001$), such that Americans generally reported lower levels of self-esteem. We found no other effects of situational or dispositional factors.

2.2 Other dependent measures from daily diary

2.2.1 Stress.

Higher levels of stress were associated with lower degrees of fulfillment across countries (B ± S.E. = -8.155 ± 1.264, 95% CI = [-10.656, -5.648], b = -0.332, $t = -6.452$, $p < 0.001$). However, we observed no difference in self-reported stress between countries nor an interaction between country and fulfillment.

Perceived legitimacy was a significant predictor of stress across countries (B ± S.E. = 9.685 ± 3.596, 95% CI = [2.63, 16.67], b = 0.297, $t = 2.693$, $p = .007$). There was also an interaction between legitimacy and fulfillment, such that not fulfilling a highly legitimate expectation is associated with more stress (B ± S.E. = -2.694 ± 1.023, 95% CI = [-4.682, -0.689], b = -0.57, $t = -2.633$, $p = .009$). Perceived strength of the expectation did not directly influence stress, but there was a marginal interaction between country and strength such that American participants were more sensitive to the effects of strength in their response to stress, in comparison to Chinese participants (B ± S.E. = 13.854 ± 7.197, 95% CI = [-0.311, 27.821], b = 0.777, $t = 1.925$, $p = .055$). Feelings of closeness and the dispositional factors of family achievement guilt and individualism-collectivism had no effect on stress.

2.2.2 Self-esteem.

Feelings of self-esteem were positively related to degree of fulfillment (B ± S.E. = 2.856 ± 1.003, 95% CI = [0.90, 4.84], b = 0.127, $t = 2.849$, $p = .005$) across countries. American participants were generally more likely to report lower levels of self-esteem (B ± S.E. = -35.328 ± 6.562, 95% CI = [-48.078,-22.437], b = -0.529, $t = -5.304$, $p < .001$) and there was also a

significant interaction between country and fulfillment, such that American participants were more sensitive in their response to degree of fulfillment (B ± S.E. = 6.725 ± 1.743, 95% CI = [3.27, 10.12], b = 0.381, $t = 3.858$, $p < .001$).

We found no influence of legitimacy or strength on self-esteem. There was an interaction effect between closeness and fulfillment (B ± S.E. = 1.808 ± 0.841, 95% CI = [0.163, 3.451], b = 0.381, $t = 2.15$, $p = .032$) such that those who feel closer to the source of the expectation are more likely to report higher self-esteem upon fulfilling the expectation.

We found no significant effect of family guilt on self-esteem. However, we found an interaction between degree of individualism and country such that more individualistic American participants had increased self-esteem, but the opposite is true for Chinese participants (B ± S.E. = 14.831 ± 7.129, 95% CI = [1.059, 28.865], b = 0.273, $t = 2.08$, $p = .039$).

2.3 Bayesian Robustness Check

2.3.1 Model specification.

We fitted a Bayesian linear mixed model (estimated using MCMC sampling with 4 chains of 2000 iterations and a warmup of 1000) to predict our outcome variables of primary negative affect and behavior change using the same predictors as before. The model also included fulfill as a random slope and subject ID as a random effect. For models assessing the predictive power of situational factors, they included one of the situational factors as an additional predictor along with its interaction with country and fulfillment. Priors over parameters were all set as normal

distributions. Following the Sequential Effect eXistence and sIgnificance Testing (SEXIT) framework, we report the median of the posterior distribution and its 95% CI (Highest Density Interval), along the probability of direction (pd), the probability of significance and the probability of being large. In line with the standards for linear models, the thresholds beyond which the effect is considered as significant (i.e., non-negligible) and large are $|0.05*SD_y|$ and $|0.3*SD_y|$.

2.3.2 Guilt.

The model's explanatory power was substantial (R2 = 0.50, 95% CI[0.42, 0.56], adj. R2 = 0.29) and the part related to the fixed effects alone (marginal R2) is 0.24 (95% CI [0.17, 0.32]). Within this model, the effect of fulfill (Median = -6.65, 95% CI [-8.88, -4.53]) has a 100.00% probability of being negative, 100.00% of being significant (< -1.49), and 2.30% of being large (< -8.92). The effect of country [US] (Median = 15.12, 95% CI [-0.09, 30.27]) has a 97.45% probability of being positive, 96.03% of being significant (< -1.49), and 79.30% of being large (< -8.92). The effect of fulfill × country [US] (Median = -3.42, 95% CI [-7.12, 0.33]) has a 96.50% probability of being negative, 84.62% of being significant (< -1.49) and 0.22% of being large (< -8.92).

Looking at the situational factors, the model including legitimacy has an explanatory power of (R2) 0.39 (95% CI [0.31, 0.46], adj. R2 = 0.29) and the part relating to the fixed effects alone (marginal R2) was of 0.26 (95% CI [0.19, 0.33]). The effect of legitimacy (Median = 11.27, 95% CI [5.30, 17.00]) has a 100.00% probability of being positive, 99.98% of being significant (> 1.50), and 77.68% of being large (> 9.00). The effect of legitimacy × fulfill (Median = -2.70, 95% CI [-4.49, -0.91]) has a 99.88% probability of being negative, 90.88% of

being significant (< -1.50), and 0.00% of being large (< -9.00). The effect of legitimacy × country [US] (Median = 8.18, 95% CI [-1.98, 18.67]) has a 94.08% probability of being positive, 89.95% of being significant (> 1.50), and 43.23% of being large (> 9.00). The effect of legitimacy × fulfill × country [US] (Median = -2.94, 95% CI [-5.91, 0.09]) has a 97.20% probability of being negative, 82.23% of being significant (< -1.50), and 0.00% of being large (<-9.00).

Moving next to strength, the model's explanatory power is substantial (R2 = 0.33,95% CI [0.26, 0.41], adj. R2 = 0.25) and the part related to the fixed effects alone (marginal R2) is of 0.20 (95% CI [0.14, 0.25]). Within this model, the effect of strength (Median = 4.38, 95% CI [-2.58, 11.88]) has a 89.00% probability of being positive, 79.33% of being significant (> 1.49), and 11.55% of being large (> 8.92). The effect of strength × fulfill (Median = -0.90, 95% CI [-3.04, 1.10]) has a 80.25% probability of being negative, 27.68% of being significant (< -1.49), and 0.00% of being large (< -8.92). The effect of strength × country [US] (Median = 6.96, 95% CI [-4.63, 18.38]) has a 87.58% probability of being positive, 81.97% of being significant (> 1.49), and 37.10% of being large (> 8.92). The effect of strength × fulfill × country [US] (Median = -0.94, 95% CI [-4.03, 2.23]) has a 72.00% probability of being negative, 37.00% of being significant (< -1.49), and 0.00% of being large (<-8.92).

The model with closeness had an explanatory power of 0.48 (R2 = 0.48, 95% CI [0.41, 0.56], adj. R2 = 0.27) and the part related to the fixed effects alone (marginal R2) is 0.27(95% CI [0.19, 0.34]). The effect of closeness (Median = 1.33, 95% CI [-5.49, 8.01]) has a 65.40% probability of being positive, 48.08% of being significant (> 1.50), and 1.32% of being large (> 9.00). The effect of closeness × fulfill (Median = -0.26, 95% CI [-2.08, 1.51]) has a 61.45%

probability of being negative, 8.15% of being significant (< -1.50), and 0.00% of being large (< -9.00). The effect of closeness × country [US] (Median = -12.34, 95% CI [-23.44, -0.90]) has a 98.25% probability of being negative, 96.80% of being significant (< -1.50), and 71.65% of being large (< -9.00). The effect of closeness × fulfill × country [US] (Median = 2.63, 95% CI [-0.28, 5.50]) has a 96.20% probability of being positive, 77.45% of being significant (> 1.50), and 0.00% of being large (> 9.00).

2.3.3 Depression.

The base model's explanatory power is substantial (R2 = 0.68, 95% CI [0.62, 0.72], adj. R2 = 0.49) and the part related to the fixed effects alone (marginal R2) is 0.28 (95% CI [0.19, 0.38]). The effect of fulfill (Median = -4.40, 95% CI [-6.41, -2.35]) has a 100.00% probability of being negative, 99.83% of being significant (< -1.36), and 0.00% of being large (<-8.17). The effect of country [US] (Median = 20.81, 95% CI [4.77, 36.63]) has a 99.48% probability of being positive, 99.08% of being significant (> 1.36), and 94.65% of being large (> 8.17). The effect of fulfill × country [US] (Median = -3.88, 95% CI [-7.51, -0.34]) has a 98.32% probability of being negative, 92.07% of being significant (< -1.36), and 1.07% of being large (< -8.17).

Moving next to legitimacy, the model's explanatory power is slightly weaker (R2 = 0.38, 95% CI [0.29, 0.46], adj. R2 = 0.28) and the part related to the fixed effects alone (marginal R2) is of 0.18 (95% CI [0.12, 0.24]). The effect of legitimacy (Median = 1.77, 95% CI [-3.31, 7.06]) has a 75.75% probability of being positive, 56.50% of being significant (> 1.36), and 1.03% of being large (> 8.17). The effect of legitimacy × fulfill (Median = -1.48, 95% CI [-3.11, 0.10]) has a 96.65% probability of being negative, 55.90% of being significant (< -1.36), and 0.00% of

being large (< -8.17). The effect of legitimacy × country [US] (Median = 6.53, 95% CI [-1.64, 14.76]) has a 94.15% probability of being positive, 89.08% of being significant (> 1.36), and 35.15% of being large (> 8.17). The effect of legitimacy × fulfill × country [US] (Median = -1.50, 95% CI [-4.10, 1.09]) has a 87.38% probability of being negative, 54.20% of being significant (< -1.36), and 0.00% of being large (<-8.17).

The model pertaining to strength had an explanatory power (R2) of 0.68 95% CI [0.63, 0.73], adj. R2 = 0.50) and the part related to the fixed effects alone (marginal R2) is of 0.33 (95% CI [0.23, 0.42]). The effect of strength (Median = 2.52, 95% CI [-3.96, 9.34]) has a 76.58% probability of being positive, 63.12% of being significant (> 1.36), and 4.95% of being large (> 8.17). The effect of strength × fulfill (Median = -0.63, 95% CI [-2.38, 1.00]) has a 75.58% probability of being negative, 19.80% of being significant (< -1.36), and 0.00% of being large (< -8.17). The effect of strength × country [US] (Median = 14.91, 95% CI [3.70, 25.31]) has a 99.50% probability of being positive, 99.02% of being significant (> 1.36), and 86.98% of being large (> 8.17). The effect of strength × fulfill × country [US] (Median = -2.46, 95% CI [-5.09, 0.21]) has a 96.03% probability of being negative, 77.78% of being significant (< -1.36), and 0.00% of being large (< -8.17).

The closeness model's explanatory power is substantial (R2 = 0.69, 95% CI [0.63, 0.74], adj. R2 = 0.50) and the part related to the fixed effects alone (marginal R2) is 0.34 (95% CI [0.25, 0.44]). The effect of closeness (Median = 2.96, 95% CI [-2.36, 8.76]) has a 86.05% probability of being positive, 72.45% of being significant (> 1.33), and 4.52% of being large (> 8.00). The effect of closeness × fulfill (Median = -1.52, 95% CI [-3.02, -0.15]) has a 98.55% probability of being negative, 59.85% of being significant (< -1.33), and 0.00% of being large (<

-8.00). The effect of closeness × country [US] (Median = -10.71, 95% CI [-21.20, -0.50]) has a 97.95% probability of being negative, 96.60% of being significant (< -1.33), and 70.70% of being large (< -8.00). The three-way interaction between closeness, fulfillment, and country [US] (Median = 2.33, 95% CI [-0.08, 4.96]) has a 97.05% probability of being positive, 78.53% of being significant (> 1.33), and 0.00% of being large (> 8.00).

2.3.4 Behavior change.

The model's explanatory power (R2) was 0.52, (95% CI [0.42, 0.61], adj. R2 = 0.30) and the part related to the fixed effects alone (marginal R2) is of 0.33 (95% CI [0.23, 0.42]). The effect of fulfill (Median = 0.40, 95% CI [0.27, 0.52]) has a 100.00% probability of being positive, 100.00% of being significant (> 0.06), and 71.55% of being large (> 0.36). The effect of country [US] (Median = -0.16, 95% CI [-0.88, 0.51]) has a 67.27% probability of being negative, 60.75% of being significant (< -0.06), and 29.45% of being large (< -0.36). The interaction between country and fulfillment (Median = -0.26, 95% CI [-0.46, -0.03]) has a 98.67% probability of being negative, 95.40% of being significant (< -0.06), and 16.57% of being large (< -0.36).

The legitimacy model has an explanatory power (R2) of 0.48 (95% CI [0.41, 0.55], adj. R2 = 0.43) and the part related to the fixed effects alone (marginal R2) is of 0.47 (95%CI [0.41, 0.54]). The effect of legitimacy (Median = 0.70, 95% CI [0.46, 0.95]) has a 100.00% probability of being positive, 100.00% of being significant (> 0.06), and 99.67% of being large (> 0.36). The effect of legitimacy × fulfill (Median = -0.02, 95% CI [-0.11, 0.07]) has a 67.95% probability of being negative, 18.73% of being significant (< -0.06), and 0.00% of being large (< -0.36). The effect of legitimacy × country [US] (Median = 0.11, 95% CI [-0.28, 0.47]) has a 71.35%

probability of being positive, 60.25% of being significant (> 0.06), and 8.97% of being large (> 0.36). The three way interaction between legitimacy, fulfillment, and country [US] (Median = -0.15, 95% CI [-0.29, 4.10e-03]) has a 96.97% probability of being negative, 86.55% of being significant (< -0.06), and 0.18% of being large (< -0.36).

Looking next at strength, the model has an explanatory power (R2) of 0.26, (95% CI [0.22, 0.37], adj. R2 = 0.22) and the part related to the fixed effects alone (marginal R2) is of 0.28 (95% CI [0.21, 0.34]). The effect of strength (Median = 0.24, 95% CI [-0.10, 0.56]) has a 91.72% probability of being positive, 84.65% of being significant (> 0.06), and 22.88% of being large (> 0.36). The interaction between strength and fulfill (Median = 0.01, 95% CI [-0.10, 0.13]) has a 57.98% probability of being positive, 21.00% of being significant (> 0.06), and 0.00% of being large (> 0.36). The effect of strength × country [US] (Median = 0.09, 95% CI [-0.43, 0.62]) has a 62.88% probability of being positive, 53.80% of being significant (> 0.06), and 16.62% of being large (> 0.36). Lastly, the effect of strength × fulfill × country [US] (Median = -0.03, 95% CI [-0.22, 0.15]) has a 63.80% probability of being negative, 39.57% of being significant (< -0.06), and 0.05% of being large (< -0.36).

The model with closeness had an explanatory power (R2) of 0.53 (95% CI [0.44, 0.62], adj. R2 = 0.30) and the part related to the fixed effects alone (marginal R2) is of 0.35 (95% CI [0.26, 0.44]). The effect of closeness (Median = -0.02, 95% CI [-0.34, 0.32]) has a 54.00% probability of being negative, 40.27% of being significant (< -0.06), and 2.08% of being large (< -0.36). The effect of closeness × fulfill (Median = 0.04, 95% CI [-0.06, 0.14]) has a 80.38% probability of being positive, 37.30% of being significant (> 0.06), and 0.00% of being large (> 0.36). The effect of closeness × country [US] (Median = -0.17, 95% CI [-0.68, 0.36]) has a

74.67% probability of being negative, 67.00% of being significant (< -0.06), and 23.62% of being large (< -0.36). And finally, the three-way interaction between closeness, country, and fulfillment (Median = 0.02, 95% CI [-0.15, 0.18]) has a 61.02% probability of being positive, 32.38% of being significant (> 0.06), and 0.00% of being large (> 0.36).

2.4. Situational factors as a function of country

We ran a separate set of linear mixed effects models with the situational factors as the dependent variable and country as the predictor variable, to understand whether the ratings of the situational factors varied systematically across countries. We controlled for the same demographic variables as before, including the participants' age, socioeconomic status, sex, and the number of siblings, and also included subject ID as a random factor.

We found that the American participants judged the expectations they reported as weaker (B ± S.E.= -0.264 ± 0.112, 95% CI = [-0.482, -0.046], b = -0.132, t = -2.352; p = .02) and less legitimate (B ± S.E.= -0.45 ± 0.12, 95% CI = [-0.677, -0.224], b = -0.201, t = -3.857; $p < 0.001$) than the Chinese participants. Overall, the American participants reported feeling less close to the sources of their expectations than the Chinese participants (B ± S.E.= -0.423 ± 0.161, 95% CI = [-0.74, -0.11], b = -0.153, t = -2.628; p = 0.009).

2.5 Mediation analysis

It has been theorized and empirically demonstrated that guilt motivates the agent to make amends and compensate the victim (Baumeister et al., 1994; Tangney et al., 2007; de Hooge et al., 2007, 2011, 2019; Yu et al., 2014). To explore the relationship among expectation fulfillment, guilt, and behavior change, we conducted a mediation analysis with the aim of

understanding the role of negative affect in this relationship. We reverse-coded fulfillment for ease of interpretation, and refer to this variable as degree of violation, with greater values signifying greater violation of the expectation. We conducted two MCMC (Markov Chain Monte Carlo) mediation models (10,000 simulations following PROCESS MACRO model 4, implemented by the bruceR package; Hayes, 2018) with either guilt or depression as the mediator in the relationship between violation and behavior change. We included subject ID as a random factor, as well as country demographic variables as covariates in this relationship.

In the model with guilt as the mediator, we found that the total effect (c) of violation predicting behavior change was significant (B ± S.E.= -0.316 ± 0.047, MCMC 95% CI = [-0.409, -0.224], $z = -6.659$; $p < .001$). Similar to our previous findings, guilt was positively associated with degree of violation of the expectation (path a; B ± S.E.= 4.983 ± 1.307, $t = 3.183$; $p < .001$). Interestingly, guilt was also positively associated with behavior change (path b; B ± S.E.= 0.006 ± 0.002, $t = 3.366$; $p < .001$) such that greater guilt was associated with an increased desire to alter behavior to fulfill expectations. Overall, the indirect (path ab; B ± S.E.= 0.031 ± 0.012, MCMC 95% CI = [0.011, 0.057], $z = 2.567$; $p = .01$) and direct effect (path c'; B ± S.E.= -0.346 ± 0.047, MCMC 95% CI = [-0.439, -0.253], $z = -7.327$; $p < .001$) of degree of violation on behavior change were significant. As a comparison, we ran a similar model with depression as the mediator but found no significant indirect effects, suggesting that the negative affect of guilt is more directly related to behavior change. It should be noted that the mediation results are correlational and should be interpreted with caution.

2.6 Descriptive Statistics

We found that Chinese participants had a higher proportion of completed responses where an expectation was experienced and reported than the U.S. American participants (B ± S.E. = 0.423 ± 0.092, 95% CI = [0.243, 0.605], b = 0.106, z = 4.615, p < 0.001). Of the expectations reported, as **Figure S3** shows, Chinese participants were more likely to report expectations about academic achievement (B ± S.E. = 0.304 ± 0.157, 95% CI = [0.021, 0.643], b = 0.298, z = 2.059, p = .050) and relationship investment (B ± S.E. = 0.820 ± 0.181, 95% CI = [0.475, 1.186], b = 0.752, z = 4.532, p < 0.001) than American participants. In contrast, American participants were more likely to report expectations related to social norms (B ± S.E. = 1.276 ± 0.314, 95% CI = [0.673, 1.921], b = 1.712, z = 4.066, p < 0.001) than their Chinese counterparts. There were no significant differences in other types of expectations between the two groups.

As for the sources of expectations, the Chinese participants were more likely to report expectations from classmates than the American participants (B ± S.E. = 1.71 ± 0.38, 95% CI = [1.01, 2.53], b = 1.96, z = 4.49, p < 0.001; **Fig. S3B**). The groups did not significantly differ in any other respect with regard to sources of expectations.

As an exploratory analysis, we examined whether the type of expectation and source of expectation had any impact on guilt, depression, and motivation to change behaviors to better fulfill the expectations. We ran linear mixed-effects models (participant ID as random intercept) predicting guilt, depression, and behavioral change from type of expectation (dummy-coded: academic performance, relationship investment, and social norms) or source of expectation (dummy-coded: parent, friend, classmate, teacher/supervisor), degree of fulfillment, country

(U.S. vs. China), and their interactions. Age, sex, SES, and sibling status were included as covariates.

We first report the results from the expectation type regression models. For guilt, across the three most frequent categories (relationship, academic performance, social norms), average guilt did not differ by type. However, we observed meaningful interactions: (a) U.S. participants tended to report higher guilt for academic expectations than Chinese participants overall (B ± S.E. = 39.67 ± 14.25, 95% CI = [12.28, 67.49], $t = 2.78$, $p = 0.005$), and (b) failures to meet academic expectations were more tightly linked to guilt among U.S. participants than among Chinese participants (B ± S.E. = 10.49 ± 3.90, 95% CI = [3.01, 18.11], $t = 2.69$, $p = 0.007$). For depression, we did not observe any significant main effect or interaction. For behavioral change, we found a significant main effect of academic performance, such that participants overall tended to exert more effort to alter their behaviors when they reported an expectation about academic performance (B ± S.E. = 1.31 ± 0.56, 95% CI = [0.22, 2.39], $t = 2.34$, $p = 0.019$).

We then examine the effects of source of expectation on the key variables: guilt, depression, and behavioral change. For guilt, there was no main effect of source, but we observed a significant interaction between country and classmate such that the U.S. participants reported significantly higher guilt when they experienced an expectation from their classmates (B ± S.E. = 75.78 ± 26.81, 95% CI = [24.34, 127.57], $t = 2.83$, $p = 0.005$). Interestingly, we also found a significant three-way interaction (B ± S.E. = -11.09 ± 5.26, 95% CI = [-21.37, -1.02], $t = -2.11$, $p = 0.035$), such that the U.S. American (vs. Chinese) participants' guilt was more sensitive to the lack of fulfillment when the expectation was from their parents. For depression, we found both a main effect of friend and a friend-by-fulfillment interaction. Specifically,

participants overall tended to report less depression when they experienced an expectation from their friend (B ± S.E. = -23.08 ± 9.59, 95% CI = [-41.88, -4.61], $t = -2.41$, $p = 0.016$), and their depression was also less sensitive to lack of fulfillment if the expectation was from their friend (B ± S.E. = 4.80 ± 2.43, 95% CI = [0.14, 9.54], $t = 1.98$, $p = 0.048$). For behavioral change, we observed a main effect of friend, such that participants overall were less likely to make effort to better fulfill an expectation if it was from their friends (B ± S.E. = -2.09 ± 0.79, 95% CI = [-3.61, -0.58], $t = -2.66$, $p = 0.008$). We also observed country-wise differences in the following sources of expectation – parent, friend, and classmate. Across all these three sources, the U.S. American (vs. Chinese) participants were more likely to change their behavior to better fulfill the expectation (parent: B ± S.E. = 3.77 ± 1.36, 95% CI = [1.17, 6.41], $t = 2.77$, $p = 0.006$; friend: B ± S.E. = 2.95 ± 1.32, 95% CI = [0.42, 5.50], $t = 2.23$, $p = 0.026$; classmate: B ± S.E. = 4.69 ± 2.29, 95% CI = [0.32, 9.09], $t = 2.05$, $p = 0.041$). Finally, we also found a significant three-way interaction, such that the U.S. American (vs. Chinese) participants' behavioral changes were less sensitive to the degree of fulfillment when the expectation was from their parents (B ± S.E. = -1.22 ± 0.45, 95% CI = [-2.09, -0.37], $t = -2.73$, $p = 0.007$).

3. Supplementary Figures

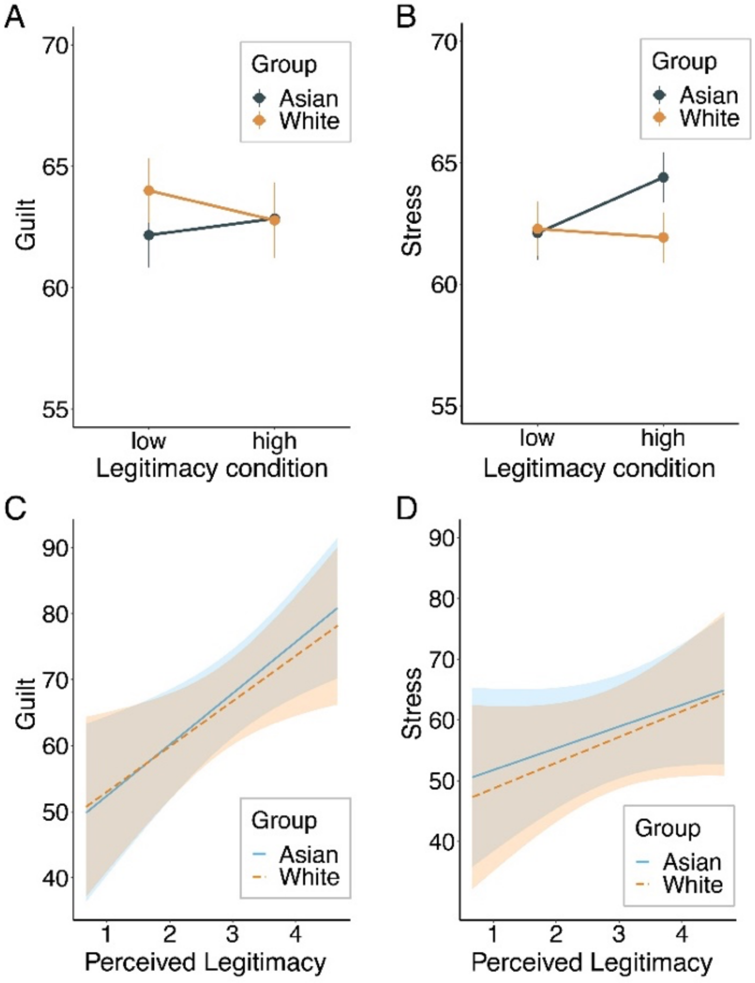

Supplementary Figure 1. Effects of legitimacy condition (A and B) and perceived legitimacy (C and D) on guilt and stress, moderated by culture.

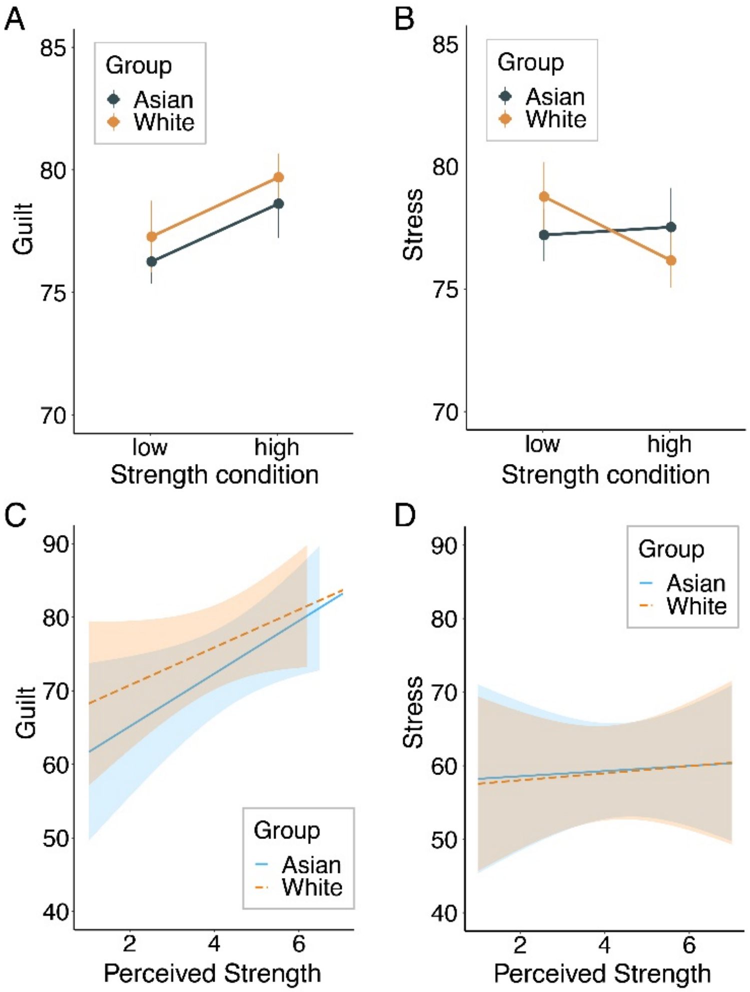

Supplementary Figure 2. Effects of strength condition (A and B) and perceived strength (C and D) on guilt and stress, moderated by culture.

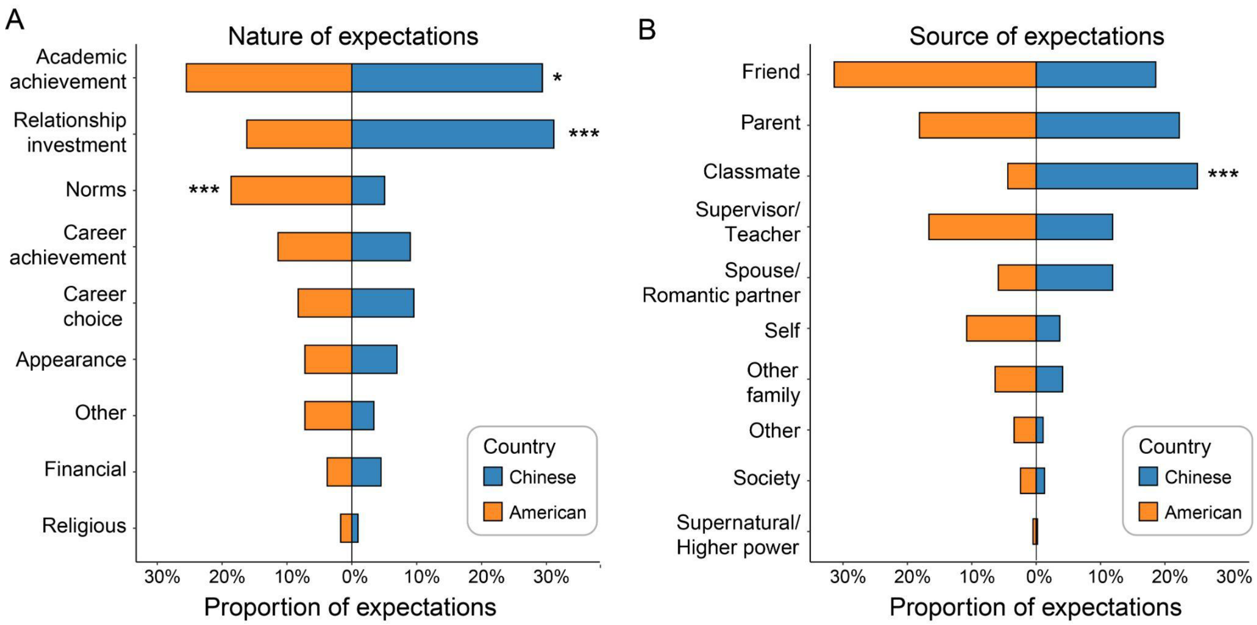

Supplementary Figure 3. Descriptive statistics of the nature (A) and source (B) of social expectations in the Chinese and the American college student sample. $^{*}p < 0.05$, $^{***}p < 0.001$.
